# Bicuspid Aortic Valve in Children and Young Adults for Cardiologists and Cardiac Surgeons: State-of-the-Art of Literature Review

**DOI:** 10.3390/jcdd11100317

**Published:** 2024-10-11

**Authors:** Francesco Nappi, Sanjeet Singh Avtaar Singh, Paolo M. de Siena

**Affiliations:** 1 Department of Cardiac Surgery, Centre Cardiologique du Nord, 93200 Saint-Denis, France; 2 Department of Cardiothoracic Surgery, Royal Infirmary of Edinburgh, Edinburgh EH16 4SA, UK; sanjeet.singh@glasgow.ac.uk; 3 Department of Cardiothoracic Surgery, Royal Brompton and Harefield Hospitals, Sydney St., London SW3 6NP, UK; paolodesiena64@gmail.com

**Keywords:** bicuspid aortic valve, aortopathy, classification, diagnosis, treatment

## Abstract

Bicuspid aortic valve disease is the most prevalent congenital heart disease, affecting up to 2% of the general population. The presentation of symptoms may vary based on the patient’s anatomy of fusion, with transthoracic echocardiography being the primary diagnostic tool. Bicuspid aortic valves may also appear with concomitant aortopathy, featuring fundamental structural changes which can lead to valve dysfunction and/or aortic dilatation over time. This article seeks to give a comprehensive overview of the presentation, treatment possibilities and long-term effects of this condition. The databases MEDLINE, Embase, and the Cochrane Library were searched using the terms “endocarditis” or “bicuspid aortic valve” in combination with “epidemiology”, “pathogenesis”, “manifestations”, “imaging”, “treatment”, or “surgery” to retrieve relevant articles. We have identified two types of bicuspid aortic valve disease: aortic stenosis and aortic regurgitation. Valve replacement or repair is often necessary. Patients need to be informed about the benefits and drawbacks of different valve substitutes, particularly with regard to life-long anticoagulation and female patients of childbearing age. Depending on the expertise of the surgeon and institution, the Ross procedure may be a viable alternative. Management of these patients should take into account the likelihood of somatic growth, risk of re-intervention, and anticoagulation risks that are specific to the patient, alongside the expertise of the surgeon or centre. Further research is required on the secondary prevention of patients with bicuspid aortic valve (BAV), such as lifestyle advice and antibiotics to prevent infections, as the guidelines are unclear and lack strong evidence.

## 1. Introduction

**Scheme 1:**
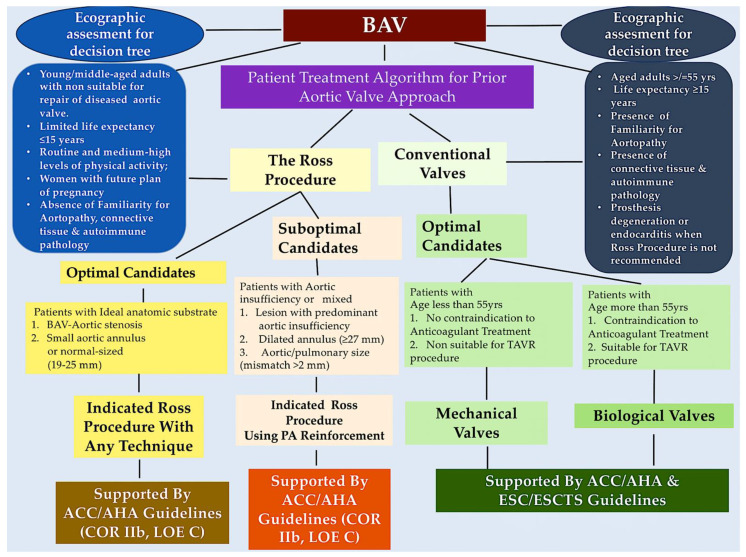
A clinical algorithm for the management of BAV based on TTE is presented herewith. COR; Recommendations for useful, effective, and beneficial indications; LOE; The level of evidence is based on moderate quality evidence from one or more well-designed, executed non-randomised studies, observational or registry studies, or meta-analyses; randomized and non-randomized observational or registry studies, meta-analyses, and physiological or mechanistic studies in humans. Abbreviations; BAV, Bicuspid Aortic Valve; ACC/AHA; American College of Cardiology/American Heart Association; COR; class of recommendation; ESC/ESCTS; European Society of Cardiology/European Society of Cardiothoracic Surgery; LOE, level of evidence.

The bicuspid aortic valve (BAV) is the most prevalent congenital heart defect in both children and adults. It can cause progressive clinical complications, including aortic valve (AV) dysfunction or aortopathy, requiring lifelong surveillance. While patients with BAV usually develop sequelae in adulthood, 12–15% of paediatric patients require intervention during childhood and adolescence [1,2,3]. In the context of BAV, assessing disease characteristics and progression in children and adolescents presents a challenge for heart disease experts. Consultations are frequently held with this population to review recommendations regarding future lifestyle choices for individuals with BAV, including participation in sports. It is essential to consider permanent surveillance and intervention times in relation to somatic growth during systematic checks. Our state-of-the-art review delves into modern research on pediatric BAV and aims to give evidence-based responses to significant clinical questions concerning the epidemiology, presentation, and management of BAV (Scheme 1).

## 2. Methods

We conducted a search of MEDLINE, Embase, and the Cochrane Library between January 2023 and March 2023, using the search terms “endocarditis” or “bicuspid aortic valve” with “epidemiology”, “pathogenesis”, “manifestations”, “imaging”, “treatment” or “surgery”. Our selection of publications was primarily from the past 20 years; however, we did not exclude older publications that were widely referenced and highly regarded. We also examined the reference lists of articles identified by this search strategy and selected pieces of literature that we deemed relevant. Recommended review articles are cited to offer readers further information and contextual references. Our analysis only encompassed articles in the English language. A breakdown of the specifics can be found in Table 1.

## 3. Prevalence of Bicuspid Aortic Valve and Epidemiological Findings

Data from global populations indicate that the prevalence of BAV is approximately 1%, with variations ranging from 0.5% to 2% depending on the populations studied and the autopsy examinations carried out [4]. Studies have shown that BAV has a lower prevalence rate and a lower risk of severe aortic valve stenosis progression in Caucasians, with greater aortic size increase compared to African Americans [5,6]. In relation to gender prevalence, the occurrence of the bicuspid aortic valve is roughly three times higher in males than in females [7,8,9,10,11]. Basso et al. [10] conducted an echocardiographic screening on primary school children and reported a prevalence of 0.5%, which was observed in 4 out of 817 children. Tutar et al. [11] confirmed these findings through an echocardiographic screening of 1075 consecutive newborns, reporting an estimated prevalence of 0.46. Larsson et al. conducted an evaluation on 21,417 autopsy specimens and reported a prevalence of 1.37%, with a total of 293 cases of BAV observed [12]. Another study conducted by Nistri and colleagues [13] in northeastern Italy involved 20,946 young men receiving echocardiographic examinations during their military service visit. The analysis of this extensive register revealed a 0.8% occurrence of bicuspid aortic valve disorder among enrolled subjects. The prevalence of BAV was reported to be 0.77% among newborns in Copenhagen in the recent Danish study by Sillesen et al. Furthermore, aortopathy was common in infants with BAV, suggesting that it may also be considered a fetal malformation [14].

### Patterns of Bicuspid Aortic Valve: Anatomy and Nomenclature

The bicuspid aortic valve has a distinctive anatomical feature where two cusps of the aortic valve AV are fused, resulting in the absence of a raphe indicating the fused commissure. Over the past 40 years, several classification schemes have been reported, allowing cardiologists and cardiac surgeons to classify BAV based on its morphological phenotype [15,16,17,18]. One commonly followed classification, described by Michelena and colleagues [17], identifies BAV by cusps involved in anatomical fusion or the highlighted raphe, as well as the underdevelopment of the remaining commissure. This results in fusion of the right-left (RL), right-non-coronary (RN), and left-non-coronary (LN) cusps, followed by corresponding underdevelopment of the third cusp.

The RL cusp fusion subtype is the most frequently observed within this phenotypic anatomy, occurring in around 70% of subjects. Subsequently, the subtype characterized by the fusion of the RN cusp was found in 28% of cases. The fusion of the cusp recognized as LN is the rarest, occurring in only 2% of patients with BAV [19,20]. In certain cases of BAV, it may not be possible to visualize a raphe at the point where the cusps join using TTE with a parasternal short or long axis view. As a result, an alternative classification has been proposed for these valves, which can be identified as anteroposterior (vertical) or lateral (horizontal) depending on the orientation of the AV opening [15]. Although this valvular anatomy can be classified as RL, RN, or LN, it refers to the typical spatial positioning of the origins of the coronary artery. In most cases, health practitioners encounter two functional asymmetrical cusps. However, it is worth noting that “true BAV” valve anatomies with circumferentially symmetrical cusps and associated sinuses have been reported [17]. The ongoing endeavour to standardise the terminology used in bicuspid aortic valve cases is apparent [19,20,21,22,23,24].

**Figure 1:**
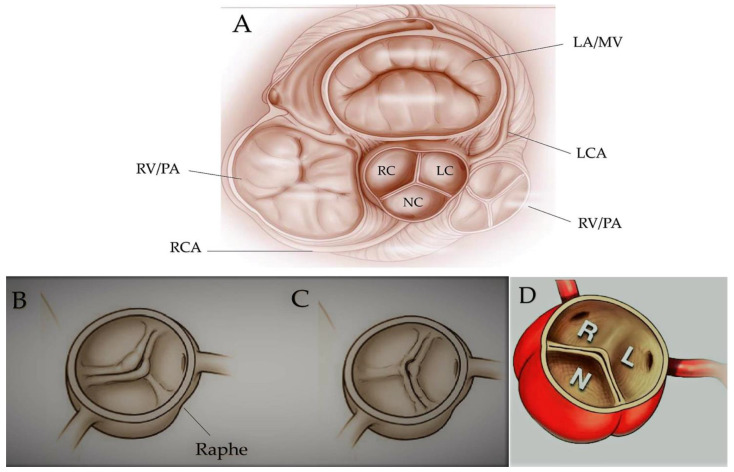
Fused bicuspid aortic valve. Panel (**A**) represents a short-axis normal tricuspid aortic pattern with anatomical proximities. Panel (**B**–**D**) represent three cusps fusion patterns seen in the short heart axis. All BAVs have three sinuses. Raphe structure is between the fused cusps. Non fused cusp is prominent in respect to the fused ones. (**B**) left-noncoronary fusion pattern; (**C**) Right-noncoronary fusion pattern; (**D**) right-left fusion pattern. The commissure angle of the non fused cusp has a degree < 180°. L, left coronary sinus; LA, left atrium; LC, left cusp; LCA, left coronary artery; MV, mitral valve; N, non-coronary sinus; NC, non-coronary cusp; PA, pulmonary artery; R, right coronary sinus; RC, right cusp; RCA, right coronary artery; RV, right ventricle. Licenses Centre Cardiologique du Nord (with permission). License Number 5644110549132 License date 8 October 2023; publication NEJM; Title: Mitral valve Repair for Mitral valve prolapse.

Assessing the BAV phenotype is essential as it helps prevent potential complications, predict future prognoses and guide follow-up. For instance, research demonstrates that patients with a fusion with the RN cusp phenotype have more significant aortic valve dysfunction and are referred for surgery more often [3]. Furthermore, the RN cusp fusion phenotype showed significantly more progression towards aortopathy with a dilated ascending aorta. In contrast, the phenotype with RL cusp fusion displayed greater dilation of the aortic root [25,26]. Furthermore, Kong et al. [27] proposed that the presence of a distinct raphe is associated with higher occurrence rates of aortic valve stenosis, aortic regurgitation, as well as greater frequency of surgical intervention on both the aortic valve and aortic conduit in the adult population. Concerns have arisen regarding the morphology of BAV, and specific genetic syndromes associated with it. RN fusion was more indicative of patients with Down syndrome, whereas in those with Turner syndrome, RL fusion was more prevalent [28,29,30,31,32,33,34,35]. Table 2 illustrates the major BAV classifications, while Figure 1 displays the varying morphology of the BAV.

## 4. Pathophysiological Features: Genetics and Histology

There is evidence from multiple studies suggesting a genetic cause for the development of BAV. Biner et al. [36] noted that aortopathy is frequently observed in first-degree relatives of patients with a bicuspid aortic valve. Fedak et al. [37] discovered that individuals with a bicuspid aortic valve were medically treated with antihypertensives to adjust their blood pressure, which maintained differences in aortic size. Siu et al. [8,38] and Tadros et al. [39] have reported that individuals with seemingly normal bicuspid valves can still exhibit echocardiographic findings such as peak aortic jet velocity and left ventricular ejection time. The significant data reveals a notable prevalence of aortic expansion in individuals with a bicuspid aortic valve exceeding the expected levels based on the parameters indicating the severity of aortic valve stenosis (AVS) or aortic valve regurgitation (AVR) [37,39,40,41,42,43]. There has been a proposition of an autosomal transmission mode with a dominant, X-linked pattern of familial inheritance [37,39,40]. Furthermore, genetic-morphological studies suggest that the migration of neural crest cells is crucial for the development of a BAV. This process leads to a common pathway in determining aortic valve disorder and aortopathy disease during the embryonic premolar stage [38,39].

Regarding the histopathological disorder, it has been observed that the fundamental structural changes are present in premolar aortopathy, which was previously known as medial cystic necrosis. Impaired regulation of the normal pathways of differentiation leads to histological abnormalities, causing the development of smooth muscle cells within the aortic tunica media. The discontinuity of the vessel wall is caused by the anomalous processing of fibrillin 1 protein present within the smooth muscle cells of the vascular endothelial element, within the extracellular matrix. Initially, smooth muscle cell detachment from the extracellular matrix is observed, which stimulates the release of matrix metalloproteinases (MMPs) and their tissue inhibitors. During the second phase, the matrix ruptures and the elastin’s lamellar organization becomes fragmented. This causes an increase in the apoptotic process within the vascular smooth muscle cells, as well as the rupture of the middle layer of the vascular conduit. These processes have an adverse effect on the structural integrity and flexibility of the aorta [37,38,39,44,45].

Wang et al. [46] analysed the plasma levels of MMPs (type 1, -2, -3, -8, -9, -10, -13) and endogenous tissue inhibitors (TIMPs-1, -2, -4) across 93 BAV patients who were grouped into four cohorts depending on the presence of valvulopathy and aortic dilatation status. The results indicated that 37 individuals showed severe AVS with ascending aorta dilatation, 28 had severe AVS without ascending aorta dilatation, 12 had normal BAV with ascending aorta dilatation, and 16 had normal BAV patients without ascending aorta dilatation. Multivariate analysis indicated that both MMP-2 and MMP-9 could be independent factors for aortic dilatation in patients with isolated severe AVS (*p* = 0.003 and *p* = 0.001, respectively). Elevated levels of MMP-2 could be a risk factor for aortic expansion in BAV patients without valvular echocardiographic abnormality (*p* = 0.002). In patients with isolated severe AS and BAV, a unique pattern of plasma MMP/TIMP was identified. This pattern could serve as a circulating biomarker for the early detection of aortic dilatation.

**Figure 2:**
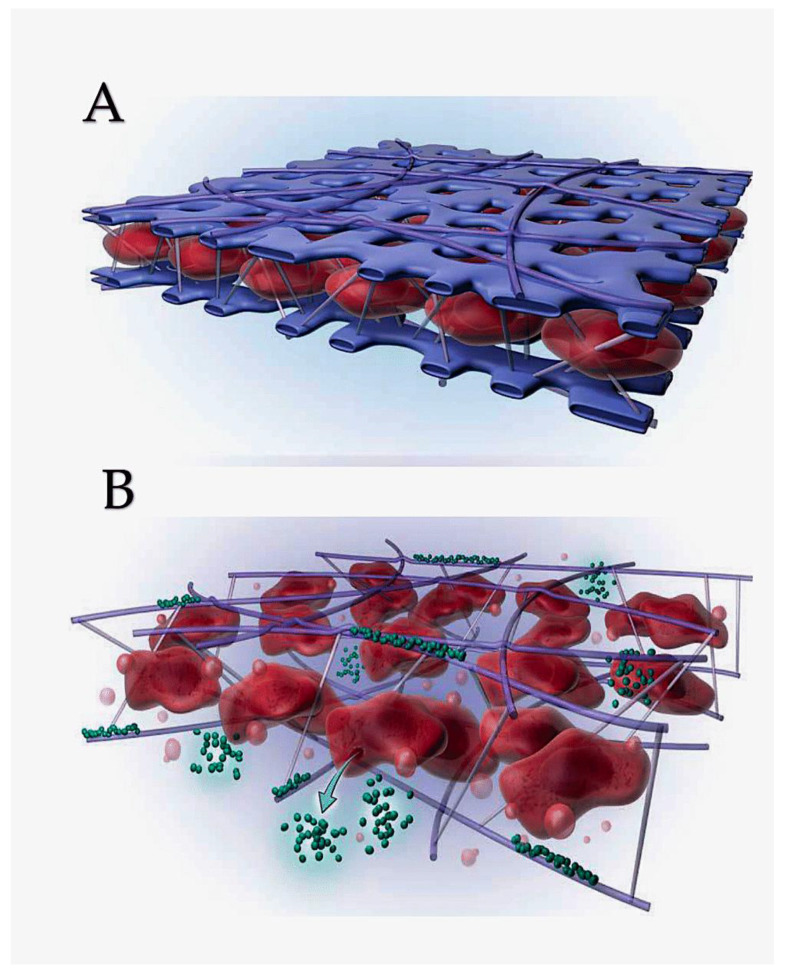
Aorta with normal morphostructure (panel (**A**,**B**) is reported. Panel (**A**): structural support and elasticity are conferred to the aorta through a morphostructure characterized by alternating layers of elastic lamellae and smooth muscle cells. Histologically, the smooth muscle cells of the aorta in individuals with tricuspid valves are attached to the adjacent elastin-collagen matrix by fibrillin 1 microfibrils. Panel (**B**): morphostructure of the aorta with bicuspid valves that may be deficient in fibrillin 1. This deficiency reaches a finale toward a disrupted morpho-architecture whereby smooth muscle cells detach, accompanied by a tide in local levels of matrix metalloproteinases (MMPs), leading to loss of integrity in the extracellular matrix and the accumulation of apoptotic cells. These events may drive to an aorta with impair structural integrity, decreased elasticity and marked tendency towards structural weakening. Licenses Centre Cardiologique du Nord (with permission); order number License Number 5663550412220; License date 7 November 2023; publication NEJM; Title: Aortic Dilatation in Patients with Bicuspid Aortic Valve.

Schmitt and colleagues [47] found that MMPs showed a more varied distribution in the aortic wall of patients with thoracic aortic aneurysm and BAV compared to those with tricuspid aortic valve morphology. BAV patients demonstrated significantly higher levels of the isoform of MMP-2, such as Pro-MMP-2 and total MMP-2. A variation in the underlying pathogenesis has been suggested due to the diverse hemodynamics affecting these subjects resulting in a heterogeneous protein level distribution. This discovery is just the beginning, and further studies are required to comprehend the pathogenetic mechanisms that promote the progression towards thoracic aortic aneurysms development, to optimize treatment and develop a screening method for its likely fatal complications (Figure 2).

## 5. Clinical Presentations and Natural History of BAV

### 5.1. Insight into Heterogeneous Clinical Phenotypes

**Figure 3:**
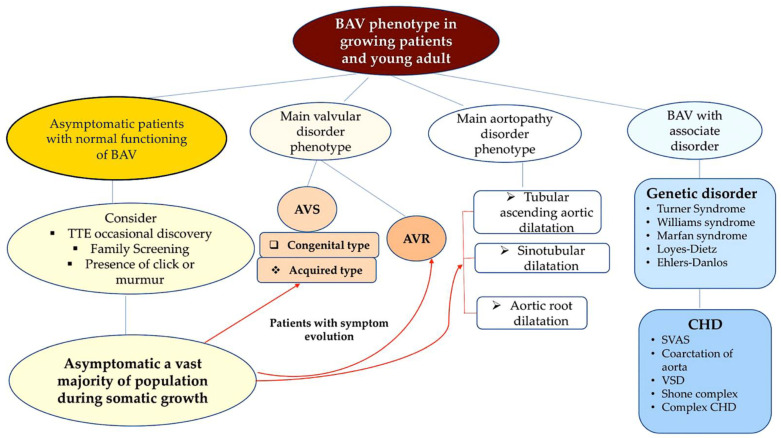
Clinical presentation and phenotypes of the BAV in patients during somatic growth and in the young adults. Various presenting phenotypes in children, adolescents, and young adults with bicuspid aortic valve are depicted in the illustrative table. The yellow box indicates the evolution of BAV in the population without AV dysfunction. The brown box illustrates the type of AV disorder while the grey box and the blue box respectively highlight the aortopathy and the disorders (genetic and congenital) that can be associated with the BAV. The red arrow indicates those patients who have aortic valve stenosis or regurgitation that manifests symptomatically during somatic growth or as young adults. AV, aortic valve; AVS, aortic valve stenosis; AVR, aortic valve regurgitation; BAV, bicuspid aortic valve; CHD, congenital heart disease; SAVS, supra-aortic valve stenosis VSD, ventricular septal defects.

Michelena et al. reported that BAV in paediatric patients can lead to three primary complications causing aortic valve dysfunction, namely aortic stenosis (AS) or regurgitation (AR), aortic dilation or development of aneurysmal disorders, ultimately culminating in bacterial endocarditis on the native aortic valve [9]. The manifestation of BAV in children varies and encompasses a broad range of clinical phenotypes, with some being linked to the age at which symptoms first appear. Refer to Figure 3 for a classification of the various clinical phenotypes, organised by their primary symptom presentation. Of note, the clinical phenotypes present may indicate the likely progression of bicuspid aortic valve disorder in adulthood for the majority of individuals affected by valve disease.

### 5.2. Imaging Diagnostic

Transesophageal echocardiography (TTE) is an essential diagnostic tool for BAV and its associated complications. Its role is especially crucial in specific cases, such as AS, where it offers invaluable insights. Studies have demonstrated the high sensitivity (78%) and specificity (96%) of TTE, along with an impressive accuracy rate of 93%. TTE is used to ascertain the morphology of the valve, the connected hemorheology, the anatomical features of the root system, the diameter and the wall alteration of the ascending aorta, as well as conditions such as the aortic coarctation associated with BAV. In the context of aortic root determination, TTE enables the measurement of the sino-tubular junction (STJ), particularly in instances of aortopathy associated with BAV [3].

The use of cardiac computed tomography (CCT) in the diagnosis of BAV is an invaluable complement to that of ultrasound scans. It is pertinent in the diagnosis of aortic dilation, the delineation of anatomical boundaries, and the correlation with adjacent structures, as well as the identification of other pathologies associated with BAV, such as aortic coarctation. Accordingly, the radiological protocol has been devised to ascertain those characteristics that may inform the surgical decision-making process, whether that entails a conventional surgical approach or transcatheter aortic valve replacement (TAVR). A 64-slice CT with a venous infusion of 50–100 mL of iodine contrast medium is typically employed for the diagnosis of BAV. It is beneficial to assess both the systolic and diastolic ECG gating phases. In the context of BAV, it is crucial to ascertain whether the true commissure or a raphe is present [3].

**Figure 4:**
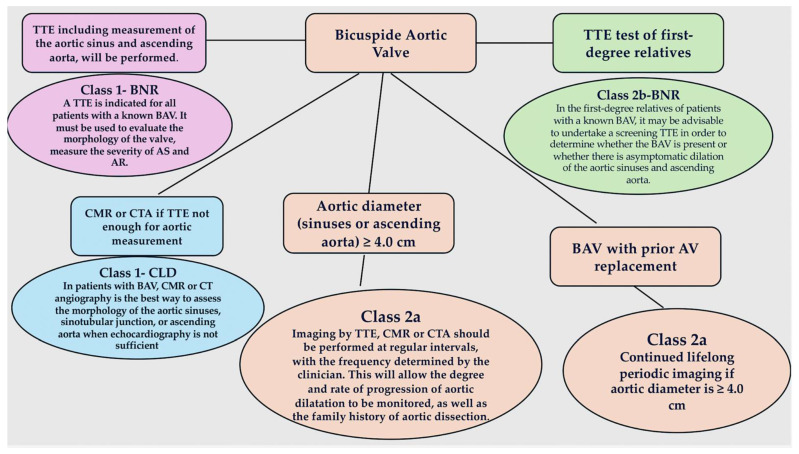
The illustration shows the recommended imaging intervals for the aorta in patients with BAV. The color of the box corresponds to the AHA/ACC guidelines for diagnostic testing (purpure, green and blue) and intervention (rose). From Nappi et al. [3,13,16,17,18,23,24].

In cases where echocardiography is unable to estimate the morphology of the aortic valve and root, as well as the diameter of the ascending aorta and arch, the contribution of the MR is of particular significance. Furthermore, it plays a supplementary role in the determination of the structure of the aortic wall and the viability of myocardial muscle. A principal function of this technique is to ascertain the extent of scar tissue within a healthy myocardium and the efficiency of cardiac chambers. It is possible to estimate the ejection fraction with this technique. It is essential to correlate these features with other decision elements derived from other imaging techniques in order to identify the most appropriate surgical indication and to forecast the prognosis for the patient. These factors render MR a more useful tool in clinical practice than CT for functional evaluation [3] (Figure 4).

### 5.3. Clinical Evidence of Asymptomatic BAV Phenotype with Normal Valve Function

The most common clinical phenotype observed in children and adolescents is asymptomatic patients with a normally functioning BAV and without clinical signs of aortic stenosis or aortic regurgitation. The diagnosis is often incidentally obtained through auscultation or echocardiography, in an otherwise healthy individual [20]. During clinical examination, a patient with a healthy BAV will display a distinctive systolic ejection click. This sound follows an S1 and can be distinguished from the normal variant, a split S1, by its lack of respiratory variability. Some patients may also exhibit a mild systolic ejection murmur [48]. The phenotype is mostly characterized by positive outcomes for a substantial number of children, who remain symptom-free during childhood and adolescence. However, some may develop aortic stenosis or aortic regurgitation. Hence, continuous follow-up and echocardiographic surveillance are advised [20,49] to monitor and manage the risk of deterioration.

### 5.4. Clinical Evidence of Aortic Valve Dysfunction Phenotype

#### 5.4.1. Aortic Valve Stenosis

Roberts and colleagues identified BAV as the predominant cause of aortic valve stenosis in both children and adults [50]. The age of onset of a BAV with clinical manifestation of aortic stenosis is variable due to differing valvular morphology and progression rates. Thus, the valvular disorder can occur from the newborn to the adolescent period. In cases of critical neonatal aortic valve stenosis, the condition may present with an associated ductal lesion that becomes dependent and occurs during the first month of life. This can lead to symptoms such as hypotension, poor peripheral perfusion, which may be associated with cyanosis, and cardiogenic shock. Given the low cardiac output, newborns may not exhibit the typical audible S3 heart murmur. Urgent intervention is necessary to address the aortic valve defect. The chosen approach, whether non-invasive balloon valvuloplasty or surgical valvotomy, is determined by the institutional preference of the reference center consulted. Importantly, several neonates may exhibit a pathological clinical presentation of variant hypoplastic left heart syndrome and may not withstand a primary repair of both ventricles [51,52].

In such instances, the recommended procedure involves mitigating the single ventricle or adopting a gradual approach to biventricular repair. AVS that occurs in childhood is non-ductal dependent, and typically presents with symptoms including progressive heart failure (HF), tachypnea, poor feeding, and failure to thrive. These individuals may exhibit a classic harsh systolic ejection murmur with or without thrill, along with an active precordium. The treatment recommendations for these patients suggest either cardiac catheterization or a standard surgical procedure on the aortic valve, prioritizing speed over urgency. It has been noted that the pathoanatomical mechanisms underlying aortic valve stenosis in infants and newborns differ from those in older patients. Neonates with critical AS often exhibit valvular thickening accompanied by partial fusion of the anterior valve commissure. Additionally, it is common to observe an anatomically unicomissural valve characterized by extensive commissural fusion, thickened leaflets, and nodular excrescences which constrict the posterior orifice [53,54,55].

In paediatric and adolescent patients with BAV and aortic stenosis, the valvular disorder progresses slowly. Somatic growth has been observed to result in a concomitant improvement in cardiac output without an increase in the effective orifice of the aortic valve. However, the adolescent growth spurt may worsen this discrepancy. The morphology of the aortic valve is marked by the presence of stiff, fibrotic leaflets. The onset of AS can be indicated by the presence of asymmetrical cusps, which restricts the size of a small, off-centre orifice. In this patient group, older children seldom seek cardiac consultation due to the absence of clear symptoms. The diagnosis is determined by auscultatory findings, particularly the presence of a harsh crescendo-decrescendo systolic ejection murmur, predominantly audible at the right upper sternal border. An increase in murmur length indicates a worsened aortic stenosis. In addition, the murmur reaches its maximum intensity later than normal, and the volume of the aortic component of the second heart sound diminishes. Two studies by Fernandes and colleagues [3,20] indicate that patients with bicuspid aortic valve morphology characterized by RN cusp fusion phenotype have significantly higher frequency and progression rate of AS and AR than those with RL cusp fusion. The valvular disorder in these subjects is characterized by the thickened, rigid fused RN cusp mechanism. Fernandes et al. [20] reported moderate or greater AS on baseline echocardiograms in 9.7% of patients with RL cusp fusion, and in 25.9% of patients with RN cusp fusion. Patients exhibiting the RN cusp fusion phenotype may require more frequent clinical observation to determine the optimal timing of surgical intervention [53,54,55].

#### 5.4.2. Aortic Valve Regurgitation

In cases of bicuspid aortic valve where a treatment procedure has not been carried out, the usual condition is that the valve leaflets will thicken and become progressively immobile. Aortic regurgitation of the trivial and mild form is not unusual, but it is rare for children to experience moderate or severe isolated aortic regurgitation. Echocardiography revealed that 33% of patients had mild aortic regurgitation, while only 4.5% of children with BAV had moderate or greater aortic regurgitation. Moreover, this group of patients had a higher mean age than the others, with 9.2 years compared to 4.1 years, respectively [20]. Fernandes et al. [1] indicated a potential progression towards more severe forms of aortic regurgitation in patients with the RN fusion phenotype when compared to those where fusion characterised the RL cusp phenotype. Fratz and colleagues [54] and Moore and colleagues [55] found that significant aortic regurgitation (AR) commonly develops in children due to a previous valve procedure, whether this involved percutaneous approach and balloon dilatation or surgical valvotomy. Although AR resulting from infective endocarditis is less common, both the degree of insufficiency and symptoms appear more severe. An initial, high-pitched decrescendo murmur heard during diastole is a noticeable feature in individuals with notable aortic regurgitation. It is most evident at the left lower sternal border and may transform with heightened severity to a holo-diastolic murmur. Patients may also exhibit an enlarged pulse pressure and bounding pulses during clinical assessment. Moderate or severe chronic aortic regurgitation in children may not show any symptoms. But, if left untreated, severe grades of AR can lead to left ventricular enlargement. This can cause symptoms of heart failure, dyspnoea, and ischemia during exertion because of coronary insufficiency. Most patients with aortic regurgitation exhibit mild, asymptomatic symptoms that remain stable for several years. Nevertheless, regular follow-ups by a paediatric cardiologist are required to monitor the progression of the disease and to decide on the optimal timing for surgery [56,57].

### 5.5. Clinical Evidence of Aortic Valve Bicuspid with Primary Aortopathy Phenotype

**Figure 5:**
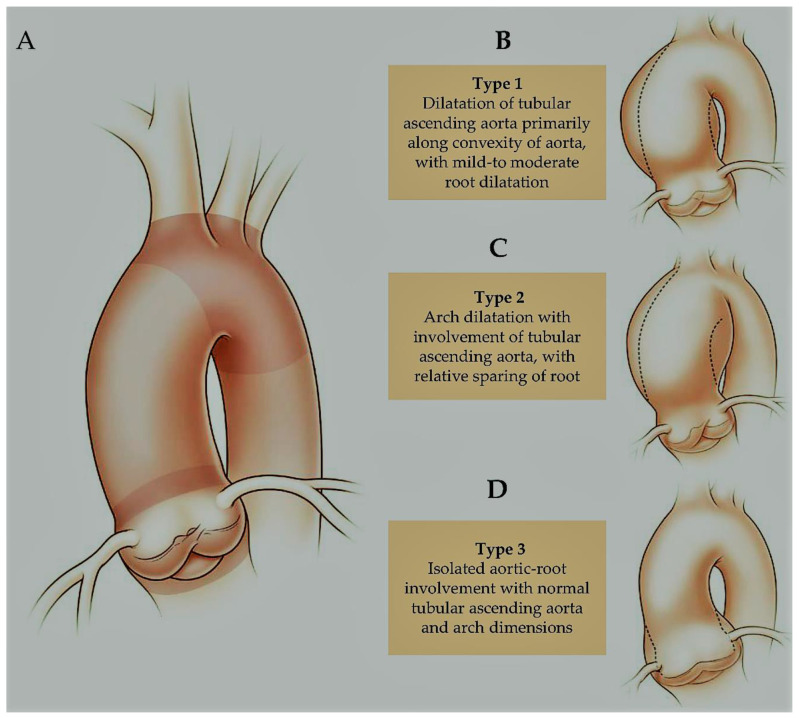
The figure discloses patterns of bicuspid aortopathy with the peculiar biologic features of the normal aorta (**A**) and the three types of bicuspid aortopathy (**B**–**D**). The three morphological types illustrated provide a crucial contribution to the best optimal surgical treatment to be performed for bicuspid aortopathy. Licenses Centre Cardiologique du Nord (with permission); order number License Number 5644101266756; License date 8 October 2023; publication NEJM; Title: Aortic Dilatation in Patients with Bicuspid Aortic Valve.

Beroukhim et al. highlighted that aortic dilatation was commonly observed in patients with BAV, including children and adolescents, without significant aortic valve dysfunction [58]. Similarly, Gurvitz et al. reported a significantly larger mean aortic size in children with BAV compared to those with a tricuspid-type aortic valve [59]. Verma et al. [38,60] and Ruzmetov et al. [61] proposed that variable patterns of aortic expansion are connected to the BAV phenotype. Several studies carried out on adult or paediatric populations have observed that the expansion of different aortic segments is dependent on the morphological BAV type. For instance, studies have found that aortic dilation is more common among patients with the RN cusp fusion phenotype, whereas those with the BAV phenotype, which exhibits fusion of the RL cusp, tend to experience a preferential expansion of the aortic root [25,26,62,63,64]. It is important to note that in patients with BAV, the main aortopathy phenotype is the characterized by ascending tubular aortic dilatation marked by sinotubular (ST) dilatation, while isolated aortic root dilatation is less common [26,64]. Numerous reports have shown ascending aortic dilatation with a Z-score greater than 2 in 49% of subjects, while aortic root expansion with a Z-score greater than 2 was reported in 11–22% of young patients [25,26]. See Figure 5 for more details.

## 6. Progression of Aortic Dilation in Growing Age, Pathogenetic Futures in Young Patients, and Spectrum of Valvuloaortopathy

In children, the dynamic nature of somatic growth can make it challenging to assess the progression of aortic dilatation. Lopez et al. [65] recommended using Z scores to body surface area as a preferred method for evaluating progressive changes in aortic dimensions and circumventing this issue. Risk factors that promote the progression of ascending aortic dilatation include an initial score of ZN2 on echocardiogram [5,64,65], RN cusp fusion morphology [51], and a higher AV gradient [25,26,58,63]. Factors found to be associated with aortic root dilation include a ZN2 score on baseline echocardiography, RL cusp fusion phenotype, significant aortic regurgitation, and male vs. female gender [25,26,62,63,65]. Patients with a bicuspid aortic valve (BAV) during infancy and adolescence may rarely develop aortic dissection without other risk factors, albeit with progressive aortic dilatation [7,26,49].

The development of BAV aortopathy is based on two theories. The first theory suggests that there is a genetic cause which supports an inherent disposition of the genetic makeup of these subjects who are susceptible to a fragility of the aortic wall. The second theory is the hemodynamic theory which is based on the accentuation of abnormal hemodynamic stress due to eccentric flow through the bicuspid aortic valve as potentially responsible for the structural disorder of the aorta [43]. Research on children strongly supports the theory that genetic factors play a role in the disease characterized by a widened aorta and a normally functioning bicuspid aortic valve. Analyzing aortic flow patterns related to aortic valve morphology and function can significantly aid the development of specific models of aortic dilatation, which can be evaluated with biomechanical modeling through finite element analysis. The progress made in genetics and the discoveries amassed through 4D flow MRI techniques are two areas of research worth investing in to gain a better understanding of the various mechanisms behind aortic dilatation and to comprehend the intricate interactions among the numerous factors responsible for the development and advancement of BAV.

**Figure 6:**
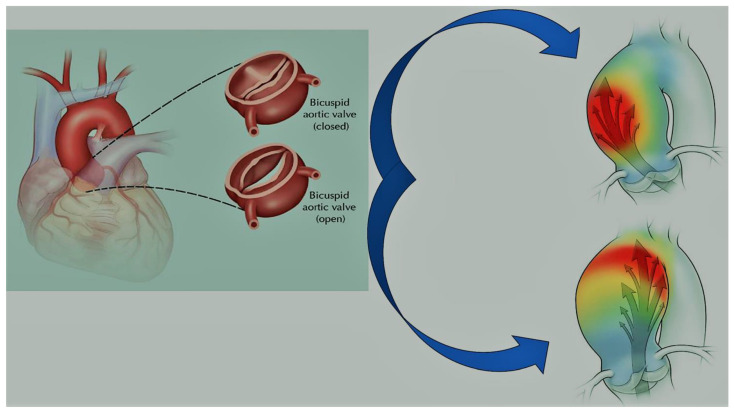
The bicuspid valve anomaly is indicated. BAV may manifest with other patho-anatomical anomalies including a morphostructural disorder of the medial tunica of the proximal ascending aorta resulting in dilatation of the ascending aorta and medial tunica of the proximal pulmonary artery. In some cases, there are changes in the coronary anatomy. The fusion configuration of the aortic valve cusps promotes changes in aortic wall shear stress and the resulting flow pattern. In the open BAV condition in the phenotype characterized by a right-left fusion, the jet is directed towards the right anterior wall of the ascending aorta. This distribution of flow travels in a right-handed helical direction causing dilation predominantly of the ascending aorta. In the fusion phenotype of the right and non-coronary cusps the jet is directed towards the posterior wall of the aorta, therefore from a biomechanical point of view the shear stress produced at the level of the can lead to aortic dilatation within the proximal arch. Licenses Centre Cardiologique du Nord (with permission); order number License Number 5655230377022; License date 8 October 2023; publication NEJM; Title: Aortic Dilatation in Patients with Bicuspid Aortic Valve.

It is common for patients to exhibit a combined valvular and aortopathic phenotype, indicating that BAV disease can have various clinical and pathological manifestations. Grattan et al. studied adolescent and adult patients who displayed aortic root dilatation, revealing a greater extent of AR [25]. Additionally, the authors observed a more pronounced post-stenotic ascending aortic dilatation in patients with severe AS due to turbulent flow directionality [25]. It is plausible to suggest that these patient groups are influenced by a more intense manifestation of BAV disease and therefore belong to a category of patients where more frequent combined aortic valve and aortic interventions are necessary (see Figure 6).

Similarly, BAV often accompanies additional congenital heart defects and genetic syndromes [28,66,67,68,69,70,71,72,73,74,75,76]. Multiple studies have identified aortic coarctation as the most frequent congenital heart defect linked with bicuspid aortic valve disease. These investigations have reported various metric discrepancies regarding BAV associated with aortic coarctation, estimating percentages ranging from 25 to 85% of patients with associated pathology. Nonetheless, the actual prevalence is likely around 30–50% [19,67,68,69]. Warnes [77] proposed that the connection between BAV and aortic coarctation may indicate two markers of a more severe pathophysiological process distinguished by diffuse arterial disease. In two separate studies, Niaz et al. [19] and Fernandes et al. [20] discovered a strong correlation between RL cusp fusion and coarctation of the aorta with a frequency of 85–90% in the phenotype that incorporated BAV plus aortic coarctation. Patients with bicuspid aortic valve (BAV) and coarctation of the aorta usually have a lower incidence of aortic stenosis or aortic regurgitation. Furthermore, patients with a history of corrected aortic coarctation who also BAV have were found to have smaller ascending aortic or aortic root dimensions and a lower prevalence of ascending aortic dilatation compared to those with isolated BAV [18]. Notably, in patients with uncorrected coarctation, BAV has been linked to an increased rate of ascending aortic dilatation [16,19,63,78,79]. Patients with BAV association and aortic coarctation have a higher incidence of intracranial aneurysms than the general population. Therefore, it may be appropriate to consider carrying out magnetic resonance angiography (MRA) or computed tomography angiography (CTA) for checks in adolescents and young adults with this disease association [79,80,81].

Turner syndrome is the genetic syndrome that is commonly linked to both bicuspid aortic valve and aortic coarctation. BAV is present in approximately 15–30% of Turner syndrome patients, while Turner syndrome occurs in an estimated 5–12% of girls diagnosed with aortic coarctation [73]. Conducting genetic screening could aid in studying these specific patient populations with BAV and aortic coarctation. The combination of BAV and aortic coarctation signals the need to investigate for phenotypic characteristics of a genetic mutation, which may often indicate Turner syndrome in girls. Additionally, due to the lack of typical features in mosaic Turner syndrome, it has been suggested that all girls presenting with coarctation undergo karyotyping [82,83]. Turner syndrome in women frequently presents a complication in the form of aortic dilatation, which raises their risk for aortic dissection significantly above that of the general population. This fact confers an additional risk factor for aortic dissection associated with BAV [73,84]. Specific measurements such as the aortic size index (ASI), which is calculated by dividing the aortic diameter by the body surface area, can be a valuable tool for evaluating the risk of aortic dissection in patients with Turner syndrome. However, the ASI is not suitable for individuals under the age of 15 due to the inconsistent ratio between aortic diameters and body dimensions. If the ASI of the ascending aorta exceeds 2.5 cm/m^2^ for women aged over 15, there may be a heightened risk of aortic dissection [73,85].

Alternative methods for evaluating the likelihood of aortic complications are practical. Such methods encompass the ascending/descending ratio of the aorta, which serves as a marker of dilatation when it exceeds 1.5. Additionally, z-scores unique to Turner syndrome [73,86] are beneficial. Some patients with Turner syndrome may have additional lesions, including BAV associated with aortic coarctation and abnormalities in the pulmonary vein connections in the atria. Due to the characteristic morphological lesions correlated with aortic expansion, magnetic resonance imaging may be a beneficial clinical tool in the diagnosis and treatment of complex pathologies in these subjects [73,87].

It should be noted that the presence of BAV can pose a substantial risk factor for aortic dilation and dissection in patients who may have different genetic connective tissue disorders, including Marfan, Loeyz-Dietz, or Ehler-Danlos syndromes [88]. Individuals with isolated dilation of the aortic root and those displaying rapid progression of aortic dilation require thorough evaluation for additional overt phenotype changes that may indicate connective tissue disorders.

## 7. Assessment and Family Screening Recommendations

BAV has been observed to manifest in three distinct modes of inheritance: autosomal dominant, X-linked, and familial [18,37,40,45] The hypothesis that abnormal migration of neural-crest cells represents a common pathway underlying the development of both bicuspid aortic valve and aortopathy has been put forth [39,45].

In children and teenagers, BAV is initially diagnosed through imaging. The recommended first step in the diagnostic workup and monitoring of progressive BAV and possible aortic dilatation is transthoracic echocardiography (TTE). The TTE is a valuable tool for comprehensively assessing the morphology of the aortic valve, outlining the different patterns of cusp fusion, and preventing complications and predicting prognosis. The parasternal short-axis view provides an easily accessible approach for exploring the morphology of the AV and detecting valvular anomalies. This approach enables us to observe the coronary arteries’ origin accurately, thus defining the location of aortic valve cusps in the sinuses of Valsalva. However, the long-axis parasternal view remains the recommended TTE approach to evaluate cusp thickness, mobility, and measure AV and aortic conduit dimensions. The American Society of Echocardiography guidelines [89] stipulate that aortic dimensions should be measured at the AV annulus, sinuses of Valsalva, ST junction, and mid-ascending aorta at end-systole, utilizing the inner edge to inner edge method.

While TTE is sufficient for evaluating the aortic root and proximal ascending aorta in younger patients, visualization of the mid-distal ascending aorta and arch can be difficult in adolescents. Therefore, a CT or MRI study should be considered for a complete evaluation of the ascending aorta in this group, especially if disease progression necessitates a surgical intervention. MRI or CT is the primary imaging modality for follow-up of older adolescents with asymmetric aortic dilatation or poor acoustic windows that make TTE unsuitable. If CT or MRI follow-up is contraindicated, a transesophageal echocardiogram may be performed. MRI is the preferred option for serial surveillance to minimize radiation exposure [90].

Maintaining a healthy diet, exercising regularly, and limiting weightlifting can help prevent the long-term progression of aortic dimensions. BAV patients should strictly adhere to these recommendations as they provide results from a widely screened population. However, despite the logic behind these measures, there is insufficient patient control data available and minimal effect of these changes [90]. Familial clustering of left heart abnormalities occurs in up to 10% of BAV patients [91,92,93]. Up to 10% of genetically screened BAV patients [91,92,93] may exhibit familial clustering of left heart abnormalities. Therefore, according to the American College of Cardiology (ACC)/American Heart Association (AHA) echocardiographic guidelines’ consensus statement, it is recommended that all first-degree relatives of the proband with BAV undergo screening to detect the presence of BAV and asymptomatic thoracic aortic disease [90,94].

## 8. Clinical Use: Mechanical Procedures of BAV in Children

### 8.1. Management of Aortic Valve with Percutaneous Intervention

In neonates presenting with critical aortic valve stenosis and depressed left ventricular systolic function, the preferred approach is balloon valvuloplasty to achieve a lower operative mortality for the index procedure, as well as a decreased risk of early reoperation [95]. A percutaneous balloon valvuloplasty that is successful can substantially postpone aortic valve surgery. However, it is common for patients to require repeat surgeries due to the added surgical complexity needed to achieve the lowest possible mortality rates. This may be due to longer operative times and heightened risks associated with extended periods of end-organ and cardiac ischemia [96]. Hill et al. [97] conducted a meta-analysis of 2368 patients from 20 studies, which reported that balloon aortic valvuloplasty (BAV) accounted for 77% of procedures, while surgical aortic valvotomy (SAV) accounted for 23%. BAV cohorts had a 46% freedom from aortic valve re-operation [95% CI 40–52], compared to 73% [95% CI 68–77] for SAV (*p* < 0.001). No significant difference was found in operative mortality (OR = 0.98, 95% CI 0.5–2.0, *p* = 0.27, I(2) = 22%) or the frequency of at least moderate aortic regurgitation at discharge (OR = 0.58, 95% CI 0.3–1.3, *p* = 0.09, I(2) = 54%) between SAV and BAV. However, D’Udekem [98] stated that SAV is better than BAV, and patients who underwent percutaneous BAV procedure had a similar regurgitation risk as those receiving open SAV repair. The previous group of patients faced a higher risk of re-intervention due to the tendency of BAV to break the valve at its thinnest and weakest part. For older children and adolescents suffering from primary AS and possessing favourable valve morphology without significant AR, percutaneous BAV may be considered as a treatment option. However, some centers prefer open SAV repair, citing superior long-term results [73]. Patients suitable for percutaneous BAV typically have severe AS, resting peak systolic gradient (via catheter) of ≥50 mmHg without symptoms or ≥40 mmHg with angina, syncope, or ST-segment abnormality at rest or during exercise [95].

### 8.2. Surgical Interventions to Manage Aortic Valve and Aortopathy

The aim of surgical correction for young BAV patients experiencing stenosis or regurgitation is to reinstate a fully functioning aortic valve. Two options are available for severe AS or AR correction due to BAV: valve replacement or valve repair.

The main benefit of AV repair is eliminating the need for the lengthy anticoagulation treatment required for mechanical valve substitutes or conventional stented bioprosthetics in accordance with international guidelines. The probability of success for a repair procedure depends on individual and institutional experience. High-volume centres have acceptable mortality rates, and a higher proportion of patients undergo aortic valve repair rather than replacement [97,98,99]. When providing guidance to families of infants and younger children, the surgeon must evaluate the probability of successful repair based on their experience and may suggest a subsequent approach. Whilst it may be probable that intraoperative conversion to aortic valve replacement will be required, the decision between the options of a biological substitute, such as a pulmonary autograft or conventional stented bioprosthetic, and the mechanical valve must be discussed with the patient’s family prior to surgery. Prior to undertaking the procedure, it is important to discuss the successful aortic valve repair, which relies on four main principles [40]. Initially, the repair must restore a suitable coaptation surface of leaflets during left ventricular diastole [17,66]. Then, the full motion of the leaflet should be reinstated or maintained. To prevent the gradual expansion of the annulus, it is advisable to use an annuloplasty ring or band to support the repair by stabilising it. In patients with coexisting connective tissue illnesses, aortic valve repair with annuloplasty reinforcement is currently being recommended. Finally, the surgeon needs to ensure that there is no more than trace-to-mild aortic regurgitation present upon completion of the repair. To our knowledge, there are also no randomized trials comparing aortic valve repair with replacement. However, observational studies indicate a benefit of AV repair at high volume centres of experience, which achieve excellent results in appropriately selected patients. With ten years of freedom from AV reoperation, 73% of patients underwent repair, while 81% received replacement [97,99].

Aortic valve replacement may involve the use of a biological substitute or a mechanical prosthesis. Nevertheless, AV replacement presents a number of disadvantages. These encompass the requirement for lifelong anticoagulation therapy and the increased likelihood of thromboembolism with traditional mechanical valves. In contrast, the utilization of traditional stented/non-stented bioprosthetic valves increases the possibility of prosthetic structural valve deterioration and failure as well as the possibility of prosthetic valve endocarditis. The Ross procedure, another AV replacement strategy of considerable merit, will be elaborated on later.

Neo-cuspidalization has emerged as a new alternative treatment option to the Ross operation and other replacement options for the aortic valve. This procedure involves reconstructing the valve’s leaflets by utilizing autologous or bovine pericardium. Although Dr. Ozaki initially described the technique for aortic valve leaflets, Acar and colleagues have been utilizing this approach since 1994 in the partial mitral homograft implantation procedure [100,101,102,103]. The Ozaki procedure has yielded satisfactory medium-term results in adult patients, but large series data for long-term outcomes is currently absent [103]. Furthermore, modifications have been made to the procedure to translate its effectiveness to the paediatric patient population [104]. However, there is currently not enough evidence to demonstrate that neocuspidization of the aortic valve is a safe and effective option for young patients who are ineligible for the Ross procedure or prefer to avoid prolonged use of anticoagulant medication [104].

Surgical treatment for aortopathy in patients with BAV involves the replacement of the aortic root or ascending aorta, with or without aortic valve preservation. It is rare for isolated dilatation of the aortic root or ascending aorta to be the primary indication for surgery in children and adolescents. The surgeon typically dictates whether aortic replacement is the appropriate treatment option when aortic valve replacement is recommended. Both adults and children adhere to the same recommendations for aortic diameter thresholds that necessitate surgery, and there are no specific indications for children. Therefore, the surgical option is determined by the patient’s clinical characteristics, comorbidities, and family history, specific to each individual [90].

According to a recent report [105], people with BAV have a high lifetime morbidity burden, with valvulo-aortopathy almost certain by the age of 90. Valvulo-aortopathy is the common clinical manifestation of BAV and is linked to extended survival. However, the presence of complex valvulo-aortopathy is connected to an increased risk of mortality. A notable portion of the population of Olmsted County, Minnesota was discovered to possess BAV via echocardiography. This equates to 652 individuals. The patients had a median age of 37 [22,23,24,25,26,27,28,29,30,31,32,33,34,35,36,37,38,39,40,41,42,43,44,45,46,47,48,49,50,51,52,53] years, comprising of 525 (81%) adults and 127 (19%) children. The cohorts include individuals with typical valvulo-aortopathy (BAV without accelerated valvulo-aortopathy or associated disorders) and those with complex valvulo-aortopathy (BAV with accelerated valvulo-aortopathy or associated disorders). The overall morbidity rate across an individual’s entire lifespan (from birth to the age of 90) was found to be 86% (95% CI 82.5–89.7). Throughout their lives, individuals experienced at least moderate aortic stenosis or regurgitation, aortic valve surgery, aortic aneurysm of ≥45 mm or z-score of ≥3, aortic surgery, infective endocarditis, and aortic dissection at rates of 80.3%, 68.5%, 75.4%, 27%, 6%, and 1.6%, respectively. Notably, individuals with typical valvulo-aortopathy had similar survival rates to the age-sex-matched Minnesota population (*p* = 0.12). Out of the total patient cohort, 562 (86%) were adults with an average age of 40 years (28–55 years), and 90 (14%) were paediatric patients with an average age of 14 years (3–26 years). Nevertheless, patients with complex valvulo-aortopathy faced survival rates lower than expected. The relative risk of excess mortality was 2.25 (*p* = 0.01) [105].

### 8.3. Ross Procedure

#### 8.3.1. Current Clinical Evidence

In the paediatric population, which is closely tied to somatic growth, the Ross procedure offers numerous benefits when compared to other valve replacements for treating aortic valve disease. These advantages include superior hemodynamics, no need for oral anticoagulation or hemolysis, and increased durability [105,106,107,108,109,110,111,112,113,114,115,116,117,118,119]. The use of this procedure has become increasingly common in reference cardiothoracic centres with extensive experience and high volumes of procedures, despite its technical complexity and changes over time. Pulmonary autograft (PA) is now widely acknowledged as the best solution for congenital and acquired ailments affecting the aortic valve and left ventricular outflow tract. For patients undergoing the Ross procedure, young adults and children often attain a 16-year survival rate of roughly 90% [108,111,114,115,116,117,118,119]. This outcome is confirmed in alternative studies that show patients living for more than two decades with a life expectancy comparable to that of the general population [118,120,121]. However, the utilization of biological derivatives presents ethical dilemmas linked to the possibility of reoperation caused by the autograft’s malfunction beyond the first decade [122,123,124,125].

Current clinical evidence from large propensity-matched observational studies indicates that the average survival of patients who underwent the Ross procedure and received a pulmonary autograft for aortic valve replacement extends well into the second decade after surgery [117,118,119,120,121,126,127,128]. The Ross procedure has demonstrated superiority in numerous studies [117,118,119,120,121,126,127,128], where survival rates of patients undergoing 10-year follow-up were compared to those of a matched general population [117,118,119,120,121,126]. These reports show that the Ross cohorts outperformed patient populations receiving conventional bioprosthetic valves for aortic valve replacement [129,130,131,132,133]. In five recent cohort studies [117,118,119,120,126], PA use was found to have superior long-term clinical outcomes, even in the second postoperative decade. In three studies, it was noted that both mechanical and bioprosthetic valves had slightly higher long-term mortality rates than those reported in the matched general population when implanted in young adults and middle-aged patients [130,131,133]. Nevertheless, optimal outcomes have been demonstrated in a significant series of Ross surgeries conducted in proficient centres, where observed long-term survival rates ranged from 87% to 95% over 15 years. Additionally, the use of PA was linked to rates of freedom from reoperation that were variably estimated between 75% and 94% over 15 years [111,116,117,118,119,120,121].

Takkenberg et al. conducted a pooled meta-analysis of thirty-nine observational studies comprising 17 consecutive series encompassing both adults and children (*n* = 2610); 12 series of adult patients (*n* = 1749); and 10 series of paediatric patients (*n* = 672). Those who underwent the Ross procedure benefitted from a low incidence of long-term valve complications [134]. Low linearised rates of pulmonary artery failure (0.78% per patient-year) and structural wear and tear of the pulmonary allograft valve (0.55% per patient-year) were observed in the study. Furthermore, pulmonary autograft and homograft endocarditis were 0.26% and 0.20% per patient-year, correspondingly. Thromboembolism, bleeding, or valve thrombosis occurred in 0.36% of patients per year. When analyzing studies with a mean patient age greater than 18 years compared to studies with a mean patient age less than 18 years, the combined rates of RVOT and autograft structural/nonstructural valve deterioration were 1. 14% (with a 95% confidence interval [CI] of 0.83 to 1.57) versus 1.69% (with a 95% CI of 1.02 to 2.79) and 0.65% (with a 95% CI of 0.41 to 1.02) versus 1.66% per patient per year (with a 95% CI of 0.98 to 2.82), respectively [134].

It is noteworthy that the study results were not affected by the follow-up duration. Moreover, a meta-analysis of 872 randomly chosen young adults assessed individuals who underwent AV replacement via three distinct surgical methods: the Ross procedure (26%), AV replacement utilizing a mechanical prosthesis (54%), or a bioprosthesis (17%). The study demonstrated a marked increase in the survival rate of patients who underwent the Ross procedure, compared to those who received a mechanical AVR. Furthermore, there was no higher incidence of complications observed in patients receiving mechanical valves compared to those who received AV replacement with bioprosthetic valves. Compared to the matched general population, the Ross procedure shows comparable survival rates. However, neither the mechanical AV replacement nor the biological AV replacement exhibited similar results [114].

Poh et al. assessed 129 consecutive recipients of the Ross procedure who had a mean age of 35 years, a BAV with regurgitation, and were monitored for an average of 10 years. Results indicate an 85% freedom from reoperation at 20 years and/or greater than mild aortic regurgitation [135]. The findings demonstrate that the efficacy and safety of using pulmonary autograft with reinforcing support, such as the Ross cylinder, were confirmed even in patients with preoperative aortic regurgitation. The favorable long-lasting outcomes align with evidence from other studies [109,121,127,128,129,136,137,138,139]. Over an extended period, these comparable findings to those of implanting prosthetic AVRs demonstrate that utilizing pulmonary autograft in young patients, even in the presence of severe aortic regurgitation, may be the optimal course of action. While there is strong emerging evidence to suggest the use of pulmonary autograft is safe for children and adolescents with BAV and without hereditary aortopathy or connective tissue disease, the Ross procedure should not be used for patients with familial aortopathy or CTD. In this particular patient population, the structural disorder of connective tissue with associated dilatation may appear late, resulting in a significant failure of the autograft.

Several recent studies have found stronger evidence of a potentially higher risk of autograft dilatation in recipients of the Ross procedure who have BAV, compared to those with non-BAV phenotype aortic valve disease. This offers greater variability in terms of good late outcomes. However, the use of PA does not seem to increase the risk of reoperation in the patient population with bicuspid aortic valves when compared to those with tricuspid valve morphology [115,117,134]. The lower use of the Ross procedure in BAV patients may be due to the distinct biomechanical behavior of the bicuspid aorta phenotype in contrast to the tricuspid aorta phenotype. Nevertheless, centers with extensive series and experience revealed results regarding survival and reoperation rates at the 19-year follow-up that did not exhibit considerable differences in the BAV phenotype when compared to the tricuspid phenotype [115,117,118,119,120,121].

While the pulmonary autograft is favoured by many surgeons, with estimates of 50% to 90% reported in patients with congenital aortic valve disease or a predominantly bicuspid aortic valve, there is limited data on the interaction between these baseline characteristics and the rates of late aortopathy or dissection in large series with long-term follow-up [115,117,118,119,120,121,126,134]. Consequently, the use of this procedure is not discouraged. Caution is warranted in the presence of bicuspid aortic valve (BAV), which tends to be a varied disorder linked to an inherited aortopathy and concomitant annulo-aortic ectasia and regurgitation in a small subset of patients. Further investigation of this condition is necessary if the Ross procedure is being considered.

#### 8.3.2. Harvesting

In the Ross procedure, there are two options for implanting the PA. The first method is sub-coronary implantation, also known as the free-end technique [108,136,137,138,139]. The second is the use of a miniroot or full root option, which involves replacing the aortic root [108]. With the sub-coronary technique, only the leaflets and annulus of the pulmonary valve are removed and preserved during insertion. The Valsalva sinus that is non-coronary could be conserved when using the alternative Ross cylinder procedure. This allows for a third option for implantation [136,137,138,139].

The free-end technique is typically utilised for individuals who have attained maximum physical growth [120]. Meanwhile, the mini root procedure involves relocating the pulmonary valve to the aortic valve’s position while retaining the continuity of the pulmonary artery, hence taking it out from the right ventricle’s infundibulum and preserving its morphology entirely. The portion of the outflow tract from the right ventricle that includes the origin of the pulmonary trunk is a complex structure known as the pulmonary infundibulum. It is made up of the conical or infundibular septum that separates the pulmonary valve from the aortic and tricuspid valves.

During the period of physical growth, it is recommended to only perform the Ross procedure using the mini root technique. This approach involves preserving both the pulmonary valve and pulmonary trunk while also reimplanting the coronary ostia [108,111,113]. The proximal insertion of the PA into the aortic annulus, involving proximal suture placement, is determined by the surgeon’s preference, as is the final length of the mini root, which results in the level where the distal suture is performed. The proximal insertion of the pulmonary artery can be performed either above or below the annulus, with adequate scalloping of the muscle rim at the valve leaflet level. This approach offers the benefit of reducing the risk of the development of transvalvular gradient. The proximal stitching may employ the continuous or interrupted approach and strength can be added by using a pericardium or Teflon strip. Surgeon preference may influence the varying length of the PA root. The distal suture line can be placed at the inferior ST junction level, although many surgeons opt to preserve the full length distally to expose the vessel to heightened mechanical stress-strain phenomena [111,112,113,127,128].

The primary concern when using the pulmonary autograft and mini-root implantation is the risk of delayed dilatation of the pulmonary artery due to elevated systemic pressures on the entire root [140]. Dilatation might arise in unsupported pulmonary sinuses, aortic annulus, and ST dilatation, leading to subsequent failure of the pulmonary artery [140]. During the growth period, Horer and team detected notable dissimilarities in the pace of PA root expansion at the neo-aortic sinus level (0.5 ± 0.1/year, *p* < 0.001) and ST junction (0.7 ± 0.2, *p* < 0.001). However, there were no changes found at the annulus (0.1 ± 0.1, *p* = 0.59) during an average follow-up of 5.1 years [110,111,112,113,141,142].

External reinforcement may reduce the risk of reoperation caused by autograft failure during PA expansion. Several technical modifications have been proposed, however, no guidelines currently control their usage. While three methods are often adopted in higher-volume centres, there is currently no long-term efficacy data available for these approaches. The most frequently employed method for strengthening the aortic root externally, which is also the most natural option, entails assimilating the pulmonary artery into the patient’s aortic root. This safeguards the pulmonary artery from the adverse consequences of systemic pressure over an extended period [121,143]. More recently, a proposal has been made to employ a Dacron external reinforcement of the pulmonary artery in order to avert late dilation by either completely impacting the mini-root or partially slinging the sinotubular junction solely [144]. Regarding the reinforcement of the aortic annulus in patients with preoperative annular dilatation, although it may mitigate early dilatation of the neo-aortic annulus [111,123], the prevention of late pulmonary artery failure, which is probably due to preoperative aortic regurgitation, has not been demonstrated [145].

**Figure 7:**
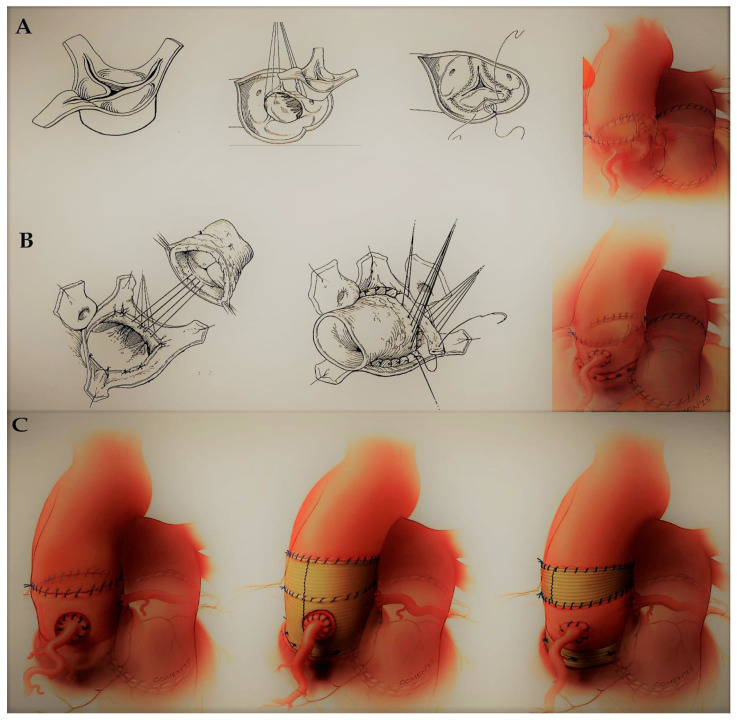
The Ross procedure is shown in Figures (**A**,**B**) with the two PA harvesting methods. (**A**): Subcoronary technique. (**B**): Full root replacement technique. Whichever technique is selected, the PA should be inserted within the annulus of the native aortic root. In Figure (**C**) technical modifications of the Ross procedure directed at mitigating late PA dilatation and failure are reported. (Right): autologous inclusion technique. (Center): dacron inclusion technique. (Left): extra-aortic annuloplasty and interposition graft. PA, pulmonary autograft. Adapted from Mazine A et al. [124,137].

We evaluated the long-term echocardiographic outcomes of the Ross procedure with and without reinforcement in 66 patients who had received aortic valve replacement using pulmonary autografts. Out of the 36 patients who underwent the non-reinforced procedure, there was an average increase in diameters of 1.28 ± 0.38 mm (3.9%) at the annulus level (in comparison with reinforced, *p* = 0.001) and 3.95 ± 0.64 mm (12.1%) at the Valsalva sinus level (in comparison with reinforced, *p* = 0.001) [104]. We noted notable variations in pulmonary autograft (PA) morpho-structure during a six-month follow-up utilizing an experimental model of the Ross operation in developing lambs. An exponential increase in the expansion of the pulmonary autograft was observed in Ross procedures reinforced with a bioresorbable vascular scaffold (BVS) made of polydioxanone (PDS) and expanded polytetrafluoroethylene (e-PTFE) as well as a semi-absorbable vascular scaffold. The index ratio for the expansion was determined to be 1.42, with dimensions of 28 ± 2 mm compared to 19 ± 2 mm and 27 ± 2 mm compared to 19 ± 2 mm, respectively. Significantly, in the Reinforced Ross procedure with a semi-absorbable vascular scaffold, the pulmonary artery behaved similarly to the normal aorta in the growing lamb. The employment of the semi-absorbable vascular scaffold, fusing polydioxanone with expanded polytetrafluoroethylene (BVS/PDS-e-PTFE), proved to be successful and secure in the pulmonary artery’s remodelling capability, while also diminishing the adverse effect of systemic pressure on the vascular wall [146,147,148,149]. The use of non-absorbable polyester reinforcement in the Ross procedure, suggested in both literature and our experimental model, may have adverse impacts on tissue vitality due to the foreign body reaction inflammatory process and the biomechanical properties of the pulmonary autograft after strengthening [150,151,152,153,154,155,156,157]. We have demonstrated the presence of macroscopic and microscopic alterations in the collected graft. The non-absorbable polyester mesh was visible and had partially migrated through the wall of the pulmonary artery, as supported by histochemical analysis [148,155,156,157] (Figure 7).

#### 8.3.3. International Guidelines and Specific Directing of Professional Societies

There is a worry that the lack of strong evidence from multicenter RCTs, when compared to observational studies, may raise doubts and lead to its recommendation being limited in international guidelines. In this instance, there may be a confounding factor associated with a type of selection bias, regardless of whether the elements have been aggregated in the research, propensity-matched data, or analyzed using multivariable methods. It is reasonable to speculate that the decision to use the PA is more likely linked to inherent features of the PA, despite being backed by efficacy and safety resulting from a meticulous patient selection. This characteristic distinguishes the pulmonary autograft due to its biomimetic nature as living tissue, accentuating its haemodynamics and biological adaptability. Despite ample evidence reporting the Ross procedure’s superiority in achieving positive long-term outcomes for aortic valve replacements compared to other surgical procedures, including data from a randomized controlled trial [126], a systematic review with meta-analyses [134], as well as multiple cohort studies supported by extensive patient series with long-term follow-up [101,117,118,119,120,121,126,127,128,137,158,159], the EAC/ESCTS continue to exclude the Ross procedure as a surgical option in class IIb recommendation [160,161]. The AHA/ACC guidelines suggest the Ross procedure as a Class IIb Level C recommendation for patients who require aortic valve replacement. However, it is emphasised that a proficient surgeon should carry out the usage of pulmonary autograft and may be an option for younger patients when VKA anticoagulant therapy is unsuitable or undesirable [126,162,163,164,165] (Table 3).

#### 8.3.4. When Use or Not to Use the Pulmonary Autograft

The use of a pulmonary autograft in the aortic position presents a weakness with regards to the extended underperformance of both aortic and pulmonary valves. This apprehension can discourage the broader implementation of this technique. Essentially, patients initially recommended for surgery due to an ailment of a single valve may necessitate additional surgery to address the affected valves. As highlighted by Stulak and colleagues [122], the surgeon executing the Ross procedure poses ethical concerns regarding the likelihood of the procedure failing or the necessity for subsequent interventions, which are prevalent.

The expansion of the conduit vessel wall, which often occurs after transposition into the left circulatory system due to the higher arterial pressure, results in decreased pulmonary valve competence and greater valvular regurgitation. This complication has a detrimental impact on left ventricular function and clinical outcomes. [111,113,116,123,140,142]. In addition, there is a potential for dysfunction of the pulmonary homograft, which is surgically implanted to reconstruct the pathway between the heart’s right ventricle and the pulmonary artery.

Based on these pathophysiological concerns, current guidelines restrict the use of PA for patients with irreparable aortic valves. The optimum method to establish the utilization of pulmonary autograft in aortic valve and/or aortic root surgery is mainly suitable for children, young or middle-aged patients below the age of 50 with other non-disabling comorbidities, who suffer from aortic stenosis, and have small or normal-sized aortic rings [165].

In young children, adolescents, and middle-aged patients, ample evidence has proven that using pulmonary autografts offers a durable solution, especially for female patients [145]. The restoration of the typical lifespan and excellent quality of life was experienced by all patient groups analyzed, with only rare valve-related complications. Conversely, managing anticoagulant medication with specific requirements, necessary for mechanical valve implantation, may pose a major challenge for women with plans for future pregnancy. It’s worth noting that utilizing biological substitutes generally obviates the requirement for long-term anticoagulant treatment [166,167]. Conversely, in young pregnant women, implementing the Ross procedure prevents the prolonged use of anticoagulant medication, thus providing an advantage in avoiding the continued danger of vascular occlusion, thromboembolism, and haemorrhage. Published evidence from numerous cohort studies, including a large population of patients who received chronic anticoagulant administration over a period of more than 20 years, indicated that patients who received conventional mechanical prostheses for aortic valve replacement incurred thromboembolic complications or major bleeding at a linearized rate ranging from 1.1% to 4.5% per patient-year [168,169].

Despite the significant advancements achieved in prosthesis manufacturing, enabling the use of new-generation mechanical valves that cause fewer thrombosis-related complications at lower international normalized ratio thresholds, self-monitoring of oral anticoagulants remains vital [170,171]. Preliminary findings suggest a reduction in the likelihood of thrombotic and bleeding complications. Nonetheless, these adverse events continue to occur as an unavoidable consequence of implementing mechanical valves.

There are certain factors that preclude the use of the Ross procedure, including preoperative AR, an aortic annulus that could potentially dilate due to a congenital morpho-structural disorder (possibly expanding to ≥27 mm), and pulmonary size mismatch [111,112,145]. These specific characteristics may compromise the longevity of the potentially at-risk pulmonary autograft, leading to gradual dilation of the aortic annulus as a result of its preexisting impaired morpho-structure. However, the aortic annulus can be reinforced through different surgical approaches, as outlined in the PA harvesting techniques section. It is possible to mitigate the impact of pressure-induced strain on connective tissue changes, which permits performing a Ross procedure on patients who have less-than-optimal conditions due to the presence of a vital anatomical structure. For example, it may be possible to reduce the gradual expansion of the implanted conduit in people with CTD or uncontrolled and extremely high blood pressures, which can have a negative impact on the functioning of the PA, by completely integrating the Ross cylinder during PA insertion [108,109,121,136,137,138,139,143]. However, for children and adolescents with structural connective tissue disorders, the benefits of the Ross procedure may be reduced. This is because enclosing the pulmonary artery (PA) in a rigid Dacron prosthesis greatly reduces its mobility, which is detrimental to the PA function as living tissue. As a result, Ross procedure recipients may experience implant failure, as indicated by a reoperation rate ranging from 20% to 50%. It is worth noting that although this is still considered a rare occurrence, it may transpire in those who are not deemed ideal candidates or for whom the use of PA is not recommended. Additionally, the incidence rate is between 1% and 2% per patient-year [118,127,134].

We hold the belief that surgeons’ habits and experience should dictate the employment of the pulmonary autograft in any aortic and root valve surgery performed on children and adolescents. This approach is founded on the intimate structure of the pulmonary artery, which is a living tissue that parallels somatic growth. Its low risk of dimensional maladaptation, coupled with its versatility in achieving efficacy and safety for interventions on the aortic valve and aortic root, as well as its perioperative safety—comparable to traditional mechanical and biological valves—renders it an ideal choice. Moreover, its demonstrated superiority as a valve replacement over traditionally utilized prostheses bolsters its potential as a viable treatment option. Even if a patient possesses a pathological bicuspid aortic valve, it should not form a reason to evade a specific treatment.

Therefore, based on the provided evidence, the possibility of opting for the Ross procedure can be discussed. It continues to be a reliable surgical recommendation with external reinforcement for young patients, children and teenagers, who have bicuspid aortic valve, and an aortic size, not exceeding 40 mm, given the absence of hereditary connective tissue or aortic disease. The choice of using the pulmonary artery as a replacement is determined by the implementation of surgical techniques that consist of adapted Ross procedures, using various external supports with the aim of stabilising the ST junction to decrease the likelihood of subsequent aortic insufficiency and dysfunction in the pulmonary artery. Overall, the interposition of the miniroot PA during surgery is determined by various factors such as evidence of comorbidities, ethical considerations, and the surgeon’s skills and experience. To improve outcomes for children and adolescents undergoing the Ross procedure and prevent potential PA dysfunction, it’s crucial to carefully consider any coexisting conditions. Hence, being mindful of these factors is essential to achieving optimal results. For example, performing the Ross procedure may not be a suitable surgical option for patients under the age of 15, those undergoing chronic dialysis due to renal failure, or those with valve disease as a result of radiotherapy. Additionally, it is worth noting that autoimmune diseases such as lupus erythematosus or rheumatoid arthritis can have an impact on the functional lifespan of the pulmonary artery. Therefore, they should be taken into account when determining the appropriate procedure. Indeed, our vast body of knowledge assists us in selecting the correct patient for PA usage and executing it flawlessly. For example, according to the current literature, there is insufficient evidence to recommend the use of PA as a viable option for patients with rheumatic disease [172,173,174]. On the other hand, the Ross procedure is a suitable alternative for patients with aortic valve endocarditis due to its low recurrence rate [105,175,176,177].

For young patients who have received pulmonary autografts in the aortic position, it is vital to secure the durability of the biological graft. These can result in the need for reoperation for pulmonary autograft dysfunction in the context of the Ross procedure. This involves searching for sensible and favourable methods to avoid undesirable issues like limited primary leaflets and the stretching of the annulus, sinuses of Valsalva and the ST junction [140,178]. A significantly high and unacceptable frequency of reoperations for PA extension has been noted in several centres [122,125,140]. Recipients of the Ross procedure who have initial pulmonary artery dilatation and experience aortic regurgitation may encounter neo-aortic root diameter enlargement upon hospital discharge. This suggests technical shortcomings inherent in the procedure [111,113,123,172,173]. Experienced Ross procedure surgeons maintain that technical improvements decrease the risk of pulmonary autograft dilatation. Implantation of PA in the intra-annular position and placing the distal suture a few millimetres below the ST junction have been demonstrated to be highly effective and safe, with excellent long-term outcomes [108,112,121,140,143,144]. This approach allows the indigenous aortic annulus to reinforce and stabilise the neo-aortic root, provided there is no preoperative dilation of the aorta or the indigenous annulus.

The possibility of malfunction of the cryopreserved pulmonary homograft (CPH) used for repairing the pulmonary-ventricular outflow tract poses a risk. The emergence of post-inflammatory valvular and supravalvular stenosis is the most common indicator of CPH failure, which predominantly occurs at the distal anastomosis [178,179,180,181,182]. Pulmonary insufficiency, on the other hand, results from rare incidences of leaflet prolapse of the allograft valve [180,182]. Preoperative pulmonary hypertension has been identified as a risk factor that exacerbates CPH dysfunction and structural degeneration. This is particularly noticeable in severe and/or irreversible cases [182]. Although pulmonary homograft dysfunction rarely results in life-threatening complications, it is important to note that the right ventricular volume and/or pressure overload are frequently endured for a significant amount of time before a reoperation involving CPH is required [111,112]. With the advent of new platforms for treating structural heart disease, patients who have suffered a failed pulmonary allograft can now undergo a transvalvular percutaneous procedure [183,184]. The two most recently available options in the market are the Melody valve (Medtronic, Dublin, Ireland) and the SAPIEN system (Edwards Lifesciences, Irvine, CA, USA).

In the Toronto series (N = 212), it was observed that using an overly large CPH reduced the risk of homograft failure after a 20-year follow-up. Although 93% of patients did not require a late reoperation as reported, echocardiography showed varying degrees of PH dysfunction. Therefore, the authors concluded that the possibility of future reoperation for these patients could not be disregarded [127]. The debate regarding the finest alternative for fixing the connection between the pulmonary trunk and the right ventricular outflow tract is worth considering. This subject has led to heated discussions, as the choice of conduit has a significant impact on its durability. Research suggests that cryopreserved pulmonary homografts are more long-lasting when used in the pulmonary position compared to cryopreserved aortic homografts [181,182]. Our study demonstrated that there were no instances of CPH failure in patients who underwent Ross procedures over a period of 23 years [111,112,113,173]. The use of CPH was prevalent for an extended period as the homograft was considered the optimal replacement option for repairing the right ventricular outflow tract [184,185].

**Figure 8:**
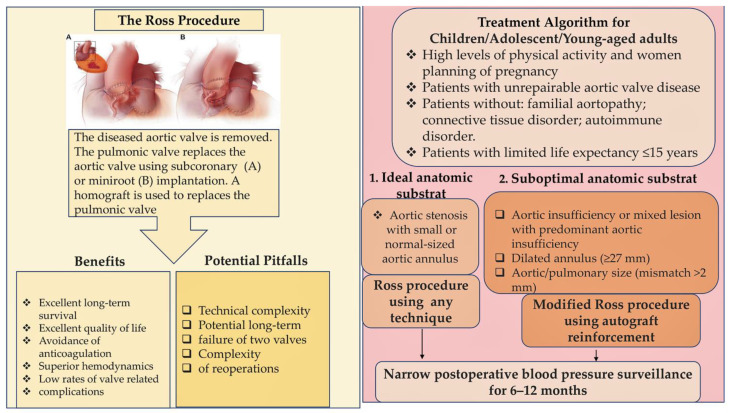
**Left**: The Ross procedure may be performed using subcoronary (**A**) or miniroot (**B**). Advantages and pitfalls of the Ross procedure are reported. **Right**: Indications and contraindications for the Ross procedure in children, adolescent and young adult patients are depicted. Patients should be referred to a high-volume referral center and referred to an experienced surgeon. This proposed algorithm requires to be further validated and supported by practice guidelines.

Recently, cardiac surgery teams have been widely adopting the use of decellularised pulmonary homografts due to their promising initial results [186]. However, it is crucial to conduct extended follow-up studies to confirm whether these derivatives will exhibit a longer duration than CPHs [146,187] and establish their usefulness. One alternative method for repairing the right ventricular outflow tract is through the use of stentless xenograft roots such as the Freestyle Porcine Aortic Root (Medtronic). Nevertheless, there is insufficient long-term data to support the efficacy of using this conduit in the pulmonary position. Please refer to Figure 8.

## 9. Clinical Use: Management of Infective Endocarditis

The lifetime incidence of infective endocarditis is higher than that of aortic dissection in patients with BAV [105]. In younger individuals, infective endocarditis impacting the aortic valve is not an often observed event in the medical and pathological sense; however, it is capable of causing substantial morbidity and mortality. According to US data from 2003 to 2010, the yearly incidence rate of infective endocarditis in juveniles was between 0.05 and 0.12 cases per 1000 hospitalisations for children [188]. It has been reported that patients with a native aortic valve (AV) and bicuspid aortic valve (BAV) have a notably higher risk of infective endocarditis when compared to the general population. Following aortic valve surgery, particularly valve replacement, patients have a higher risk of endocarditis [189,190]. The European Society of Cardiology (ESC) and the American Heart Association (AHA) guideline recommendations limit the use of endocarditis prophylaxis to high-risk conditions exclusively [188,191,192]. For most indications in guidelines, BAV is considered an intermediate-risk condition. Therefore, antibiotic prophylaxis is not recommended for patients with BAV, unless they have a history of AV surgery [188,191,192]. Therefore, antibiotic prophylaxis is not recommended for patients with BAV, unless they have a history of AV surgery [188,191,192]. Therefore, antibiotic prophylaxis is not recommended for patients with BAV, unless they have a history of AV surgery [188,191,192]. Nevertheless, a recent study proposes that prophylaxis may be necessary for patients who have a BAV disorder, particularly when there is significant AV dysfunction [92]. Patients who have received conventional mechanical or biologic prostheses or prosthetic materials as part of valve repair, undergone aortic valve replacement using a Dacron interposition graft, or have a history of endocarditis should receive endocarditis prophylaxis before undergoing high-risk procedures [191]. Furthermore, patients with BAV who develop an unexplained fever or experience complications such as embolic phenomena should be referred for a thorough clinical investigation to rule out endocarditis.

## 10. Areas of Uncertainty in Surgical Strategy

**Figure 9:**
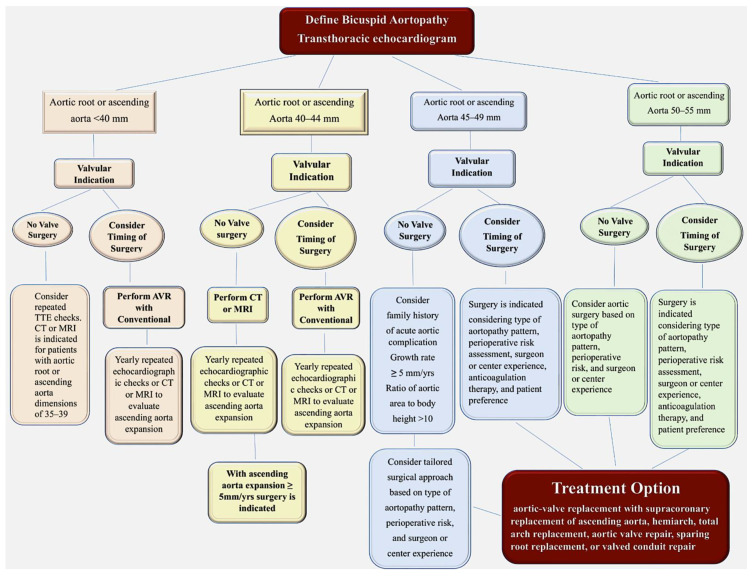
Illustrates the decision-making algorithm employed by the medical team in the management of bicuspid aortopathy. Abbreviations; AVR, aortic valve replacement; CT, computed tomography; MRI, magnetic resonance imaging. Adapted from Nappi et al. [3,53,90,160,162,197,198,199,200,201,202,203,204,205,206,207,208].

We are unaware of any randomized trials comparing aortic valve repair with aortic valve replacement in children with bicuspid aortic valve disease. It is unlikely such a trial will occur. Consequently, the current recommendation for treating severe aortic valve regurgitation in BAV disease with aortic valve repair is substantiated by observational data. It is unclear whether asymptomatic patients with severe aortic stenosis or regurgitation and no left ventricular dysfunction or dilatation or pulmonary hypertension ought to undergo early surgery. It has been noted that patients with normally functioning BAV may have impaired LV myocardial mechanics, including LV systolic, diastolic dysfunction, and LV hypertrophy. This supports the hypothesis that BAV is not only a valvular disease but also a myocardial disease [193]. If AV repair is not assured, the surgical option of AV replacement should be considered cautiously and possibly after the patient has reached 12 years of age. While some researchers have discovered indications of decreased morbidity and mortality with surgery and advise early intervention [194], others have observed that watchful waiting does not appear to result in worse outcomes [195]. If surgical intervention for AV repair is required in patients with BAV, it may be carried out with an aortic valvuloplasty to prevent the progression of annular dilatation, as stated by Youssefi et al. [196]. The surgical procedure may be performed using standard techniques or minimally invasive procedures, including ministernotomy or minithoracotomy, particularly in younger patients [160,162,197,198,199,200,201,202,203,204,205,206,207,208] (Figure 9).

The AHA/ACC guidelines advise on performing the Ross procedure for children if the success rate of surgery is expected to be over 90% [105,111,142,162,163]. Conversely, the ESC does not provide a definite recommendation for using the Ross procedure [160]. There is an increasing amount of experience with performing pulmonary autograft for BAV in newborns and children. In a multicenter series comprising of 76 infants, the mortality rate after 30 days was 17%, while the survival rate after a prolonged period was 91%. Furthermore, the freedom from autograft reintervention stood at 98% at the 10-year mark. Early fatalities were tied to neonatal age, preoperative employment of intravenous inotropic drugs, and inborn defects of the aortic arch. While these results outperform traditional methods of aortic valve repair or replacement, there is a need for further evaluation regarding its widespread applicability and cost-effectiveness, especially considering that it is currently only performed at a few specialized centers. Advances in platforms for treating structural heart disease enable a patient exhibiting pulmonary allograft failure to undergo a transvalvular percutaneous procedure [183,184].

## 11. Clinical Use: Medical Management of Aortopathy

Research into patients with genetic aortopathies, like Marfan syndrome, has influenced the medical management of BAV aortopathy with beta-blockers or angiotensin-converting enzyme (ACE) inhibitors. Various drugs have been proposed to support a range of treatment options recommended by paediatric cardiologists [209,210]. It has been suggested that beta-blockers have a positive effect on reducing aortic wall stress, resulting in a decrease in the inotropic status of the heart and a consequent reduction in heart rate and blood pressure [211]. It has been proposed that the administration of ACE inhibitors or angiotensin receptor blockers (ARBs) can inhibit transforming growth factor β (TGFβ) signaling, which promotes various genetic aortopathies [212]. However, despite the wide availability of these medications, there is presently no proof to support the beneficial role of beta-blockers, ACE inhibitors, or ARBs in aortopathies linked to bicuspid aortic valve (BAV) [213]. Multiple studies, incorporating a restricted number of patients, have suggested that beta-blockers have little or no effect on reducing ascending aortic wall sheer stress in persons with BAV [214,215,216,217]. Large longitudinal randomised controlled trials are necessary to bridge the gap in optimal pharmacological treatment for BAV aortopathy. Concerns regarding studies conducted in young patients arise because such trials demand thorough monitoring of somatic and aortic growth patterns.

## 12. Athletes and Sports Recommendations

Sports practice is a valuable recommendation for many paediatric patients diagnosed with bicuspid aortic valves. While there are no specific guidelines for younger patients considering participation in sports with this condition, referring to advice on eligibility and disqualification of athletes with cardiovascular irregularities can provide accurate recommendations [218,219,220,221]. Regular aerobic exercise should be encouraged to maintain an active lifestyle [220]. Some types of isometric exercises, such as weightlifting and wrestling, could substantially increase mean arterial pressure, thereby raising the potential risk of aortic dissection. This is a worrying development, and experts warn that people who have moderate or greater aortic dilation should steer clear of heavy isometric exercises like weightlifting. If weightlifting training is being considered for patients with BAV, it is advisable to opt for light weights for multiple sets instead of heavier weights. Providing a recommended weight limit for several athletes with BAV can offer valuable guidance, customised to their height, fitness, muscle strength, activity preferences and blood pressure response, to assess cardiopulmonary exertion [220,221].

## 13. Conclusions

Bicuspid aortic valve disease is the most common congenital heart disease. Diagnosis of BAV has various implications that necessitate continuous monitoring, with surgery advised based on severity. Several approaches exist to manage these patients, primarily to maintain valve function and avoid anticoagulation. These approaches should be decided by the proficiency of the surgeon and centre. Aortic valve repair and the Ross procedure are both surgeries with favourable long-term outcomes when performed by skilled practitioners. However, additional research is required regarding secondary prevention measures, such as providing lifestyle advice and antibiotic prophylaxis, as the guidelines surrounding these interventions are vague and lack substantial evidence.

## Figures and Tables

**Table 1:** Narrative review searching strategies. Adapted From Nappi et al. [3].

Items	Specification
Date of Search (specified to date, month and year)	From January 2024 to May 2024
Databases and other sources searched	PubMed, MEDLINE, Embase, and the Cochrane Library
Search terms used (including MeSH and free text search terms and filters)	(endocarditis” or “bicuspid aortic valve” in combination with “epidemiology”, “pathogenesis”, “manifestations”, “imaging”, “treatment”, or “surgery”)
Timeframe	Up to May 2024
Inclusion and exclusion criteria (study type, language restrictions etc.)	English language; inclusion criteria: all manuscripts on BAV treated before the age of 18; exclusion criteria: manuscripts with BAV patient cohorts older than 18 years were excluded
Selection process	Two authors independently selected articles after screening for duplicates.

**Table 2:** BAV classifications. Abbreviations; BAV, bicuspid aortic valve; BAVCon, bicuspid aortic valve consortium; LN, left non-coronary fusion; RL, right–left fusion; RN, right non-coronary fusion. Adapted From Nappi et al. [3].

Author	Nomenclature
Roberts [29] 1970	Anterior–posterior cusps Right–left cusps Presence of raphe
Brandenburg et al. [30] 1983	Clock-face nomenclature: Commissures at 4–10 o’clock with raphe at 2 o’clock (R-L) Commissures at 1–6 o’clock with raphe at 10 o’clock (RN) Commissures at 3–9 o’clock without raphe (L-N)
Angelini et al. [31] 1989	Anterior–posterior cusps Right–left cusps Presence of raphe
Sabet et al. [32] 1999	RL RN LN Presence of raphe
Sievers and Schmidtke [15] 2007	Type 0 (no raphe): anteroposterior or lateral cusps (true BAV) Type 1 (1 raphe): R-L, RN, L-N Type 2 (2 raphes): L-R, RN
Schaefer et al. [16] 2008	Type 1: RL Type 2: RN Type 3: LN Presence of raphe Aorta: Type N: normal shape Type E: sinus effacement Type A: ascending aorta dilatation
Kang et al. [18] 2013	Anteroposterior orientation: type 1: R-L with raphe type; 2: R-L without raphe Right–left orientation: Type 3: RN with raphe Type 4: L-N with raphe Type 5: symmetrical cusps with 1 coronary artery originating from each cusp Aorta: Type 0: normal Type 1: dilated root Type 2: dilated ascending aorta Type 3: diffuse involvement of the ascending aorta and arch
Michelena et al. [23] 2022	BAVCon nomenclature: Type 1: R-L Type 2: RN Type 3: L-N Presence of raphe
Jilaihawi et al. [33] 2016	Tricommissural: functional or acquired bicuspidity of a trileaflet valve Bicommissural with raphe Bicommissural without raphe
Sun et al. [34] 2017	Dichotomous nomenclature: R-L Mixed: (RN or L-N)
Murphy et al. [35] 2017	Clock-face nomenclature: Type 0: partial fusion/eccentric leaflet? Type 1: RN, RL, LN partial fusion/eccentric leaflet? Type 2: RL and RN, RL and LN, RN and LN partial fusion/eccentric leaflet?

**Table 3:** International guideline recommendations for the Ross procedure. Abbreviations; ACC, American College of Cardiology; AHA, American Heart Association; EACTS, European Association for Cardio-Thoracic Surgery; ESC, European Society of Cardiology; LOE, Level of Evidence. # Number; Change in the 2020 AHA/ACC [162].

Year Guideline (Ref. #)	Recommendation	Class of Recommendation LOE	First Author (Ref. #)
2020 AHA/ACC [162]	In patients <50 years of age who prefer a bioprosthetic AVR and have appropriate anatomy, replacement of the aortic valve by a pulmonic autograft (the Ross procedure) may be considered at a Comprehensive Valve Center [126,128,159].	2 B-NR	Buratto et al. [159] El-Hamamsy et al. [126] Martin et al. [128]
2021 ESC/ EACTS [160]	No mention of the Ross procedure	-	-

## Data Availability

Not applicable.
